# Medical students’ self-reported gender discrimination and sexual harassment over time

**DOI:** 10.1186/s12909-020-02422-9

**Published:** 2020-12-10

**Authors:** Marta A. Kisiel, Sofia Kühner, Karin Stolare, Erik Lampa, Martin Wohlin, Nina Johnston, Anna Rask-Andersen

**Affiliations:** 1 Department of Medical Sciences, Environmental and Occupational Medicine, Uppsala University, Dag Hammarskjölds Väg, 60 751 85 Uppsala, Sweden; 2 Department of Medical Sciences, Uppsala University, Uppsala, Sweden; 3 Department of Psychiatry, Uppsala University, Uppsala, Sweden; 4 UCR-Uppsala Clinical Research Center, Uppsala University, Uppsala, Sweden; 5 Department of Medical Sciences, Cardiology, Uppsala University, Uppsala, Sweden

**Keywords:** Gender discrimination, Sexual harassment, Medical students, Swedish university

## Abstract

**Background:**

Gender discrimination (GD) and sexual harassment (SH) occur at all academic institutions worldwide. Medical students report high prevalence of GD and SH, which may negatively affect their education and health. There are indications that policies and reforms on reducing GD/SH are insufficient. Swedish medical students’ experiences of GD/SH are monitored by course-evaluations and bi-annual student union evaluations; however, the response rate is usually low. The aim of this study was to compare the exposure to and context of self-reported GD/SH over an 11-year period amongst medical students at a Swedish university.

**Methods:**

In 2002, a questionnaire (*n* = 622) was mailed to medical students’ home addresses. It was repeated in 2013 and then distributed during mandatory lectures (*n* = 856). The questions used a behavioristic approach and asked about specific GH/SH experiences. Participation was voluntary and anonymous. The changes in prevalence over time were calculated by sampling weights in order to obtain comparable estimates, representative of both cohorts.

**Results:**

The response frequency was 55% (62% women) in 2002 and 81% (59% women) in 2013. The prevalence of GD tended to decrease for male and clinical students in comparison to female and pre-clinical peers. However, the prevalence of SH increased for female compared to male students. The ratio of SH for female pre-clinical students doubled in many instances; most often, the mistreatment occurred in the clinic. Medical doctors were indicated as perpetrators up to five times more often by all students in 2013.

**Conclusion:**

Our results show a disproportional change in exposure to GD/SH between female and male medical students, resulting in a widening of the gender gap regarding prevalence of GD and SH between 2002 and 2013. In particular, personal experiences of SH increased for both sexes. It is proof that institutional efforts to fight mistreatment might be ineffective.

**Supplementary Information:**

The online version contains supplementary material available at 10.1186/s12909-020-02422-9.

## Background

Gender discrimination (GD), including gender harassment, and sexual harassment (SH), occur at academic institutions worldwide [1, 2]. Of all students and academic employees, medical students report high prevalence of GD and SH [3, 4]. Although women constitute the majority of medical students, men are, still, more often in decision-making positions at medical institutions [5]. Students’ dependence on supervisors is a known risk-factor for GD and SH and facilitates potential exposure to mistreatment at medical schools. These factors affect both genders but in particular women [3–5].

A recent systematic review on international studies, the majority conducted in the United States, Canada, Pakistan, the United Kingdom, showed that 49–68% of all medical students have experienced at least one type of GD or SH during medical school and that female students are more affected than their male peers [2]. The studies underline that medical students are not overly sensitive to GD/SH [6] and may rather underreport mistreatments [7–9]. It is shown that the occurrence of GD and SH is higher in the clinical environment than in pre-clinical education [8, 10]. One proposed explanation for this is that the nature of clinical practice with, for example, examination of undressed bodies of both patients and students, contributes to the breakdown of social and sexual barriers [5, 11, 12].

The students’ acceptance of mistreatment seems to increase with the duration of the studies [10] and it is higher among junior doctors in comparison to students. It is suggested that medical students are progressively indoctrinated to accept mistreatment during medical school [13]. The socialization process of becoming a doctor is largely impacted by the clinical environment, where formal curricular frames meet reality. Coining the terms “informal” and “hidden” curriculum, an important factor for developing professional identity. The hidden curriculum is a known influencer of educational outcomes, including not openly taught norms, values and believes of the educational social environment. Much of this socialization occurs in the educational social environments. While formal instruction promotes empathy, collegiality and equality, the hidden curriculum can refuse those values and result in the acceptance of mistreatment [14, 15].

Research have shown that exposure to GD/SH in education creates an adverse learning environment for affected students but also for those observing the mistreatment of others. The experience contributes to a reduction in their confidence, learning ability, and motivation to complete their education. There are also indications of long-term negative consequences on the individual’s health, including post-traumatic stress, burnout, depression, anxiety, and chronic pain [4, 5, 12, 16].

In Sweden, the first law regarding equal treatment of students in higher education was established in 2001 [17]. It was followed by the incorporation of aspects on gender inequality in the curricula of Swedish universities. At the medical program at Uppsala University (UU), gender perspective was introduced in the syllabus as part of a larger reform in 2006. An earlier elective course on gender issues, attended by 15% of medical students, was replaced by mandatory course on gender medicine with focus on biological difference between sexes and their clinical significance. This course also included some examples of gender issues as aspects and concerns related to women’s and men’s lives and gender differences in society. Also, in 2006, problem based learning was introduced at all levels, aiming to enhance understanding of the integration between basic science and clinics [18].

Although the gender equality in higher education is considered important, there is a lack of long-term studies monitoring the prevalence of gender-based discrimination. The knowledge on medical students’ experiences of GD/SH at UU is mainly based on course-evaluations and bi-annual reports from the student unions [19, 20]; however, in both these surveys, the response rate is usually low. Therefore, the aim of this study was to evaluate whether there was a change in the prevalence and context of self-reported GD and SH among medical students at Uppsala University between 2002 and 2013.

## Methods

### The study design

This questionnaire-based study examines changes in self-reported gender discrimination (GD) and sexual harassment (SH) of medical students over an 11-year period at Uppsala University (UU) in Sweden. The first voluntary and anonymous survey was performed in 2002 and then repeated in 2013, when the new curriculum and the mandatory course in gender medicine introduced in 2006, was established [19]. The aim with waiting several years was also to have a new set of students participating in the 5,5 year long program not have the same student responded twice.

Directly after the questionnaire study was conducted, we summarized its results in Swedish and published them on the UU website [21, 22]. In the current project, we combined data from 2002 and 2013 and performed a repeated cross-sectional study [23]. We compare the results of two questionnaires with advance comparative statistics.

In accordance with Swedish law, international regulations [24, 25] and local policies at the university, gender discrimination (GD) is defined as mistreatment due to gender and includes gender harassment (a type of gender discrimination that includes a violation of someone’s dignity). Sexual harassment (SH) is defined as an act of a sexual nature that violates someone’s dignity.

The questionnaire used in this study was developed in 2002 by Werner and Grave [21] and is based on a previously published and validated survey [26]. It included questions on students’ demographics and specific examples of behaviors that constitute GD and SH (Supplementary Table 1). This behavioristic approach is shown to give the most accurate picture of self-reported experiences of mistreatment [27, 28]. Similar questionnaires have been used in international studies [29].

The Ethical Review Board in Uppsala decided that the study did not require ethical approval (DNr 2013/380).

### Data collection and study populations

All participants gave written informed consent. In 2002, all registered medical students (*n* = 689) at Uppsala University were invited to participate, and the questionnaire was mailed to the student’s home address with a request for its return. In 2013, the questionnaire was distributed to medical students in semesters 1–9 and 11 (*n* = 942) during lectures, excluding semester 10 (*n* = 67) as the students were writing their research project and spread out at the campus. In order to avoid bias, the students in the 10th semester (*n* = 57) were excluded from the cohort of 2002. The inclusion criterion in both the studies was a completed questionnaire.

The medical program at UU is a traditional program, divided into pre-clinical (semesters 1–4) and clinical semesters (semesters 5–11). In 2002, the students in semesters 1–4 mainly had pre-clinical theoretical education and only sporadic contact with the clinical environment. In 2006, a new approach was adopted, resulting in students starting their regular clinical rotations in primary care units earlier. Therefore, in 2013, medical students had contact with the clinical environment already in the first semester.

### Statistics

The students in both cohorts were stratified into groups, according to the stated sex (female/male) and current study stage (pre-clinical and clinical groups). Each student was assigned a sample weight reflecting his or her probability of inclusion, given the age and gender distributions in 2002 and 2013. The weights were further calibrated [30] to match the population totals of males and females and age groups of the different semesters in each year. Prevalence and ratios between the prevalence in the different years or between males and females were calculated by taking the sampling weights into account in order to obtain estimates that were representative of the two populations. Ratios are used as they have a natural interpretation as percent increase or decrease. While absolute difference in percentage is also useful measure of change, percentage point usually is misinterpreted as percentage [31]. Confidence intervals are Wald type confidence intervals calculated on the logit scale and back transformed to the probability scale and can thus be asymmetric. All analyses were conducted using R [32] version 3.6.1 using the survey add-on package [33, 34].

## Results

### Demographic data

In 2002, 343 (55% of all distributed surveys) medical students completed the questionnaire, 62% were women, 44% (*n* = 152) were from the pre-clinical semesters. To our knowledge, ten surveys failed to reach the student due to incorrect addresses being registered.

The response rate was higher in 2013, and a total of 720 individuals (84% of distributed surveys) returned the completed questionnaire, 59% were women, 46% (*n* = 329) students from the pre-clinical course. Table 1 shows the detailed demographic characteristics.

**Table 1:** Total number of medical students that completed the questionnaire in 2002 and 2013, stratified by pre-clinical and clinical semesters, age, year, and gender. Percent (%) is the percent of all females and males or the percent of the total of medical students in the cohort from 2002 and 2013

Semester	Age	2002			2013		
		Females n (%)	Males n (%)	Total n (%)	Females n (%)	Males n (%)	Total n (%)
**Pre-clinical**	< 20	4 (2)	1 (1)	5 (1,5)	24 (6)	23 (8)	47 (6,5)
	20–25	75 (36)	30 (22)	105 (30,5)	153 (36)	89 (30,5)	242 (33,5)
	26–30	15 (7)	12 (9)	27 (8)	11 (2,5)	15 (5)	26 (4)
	31–35	7 (3)	5 (4)	12 (3,5)	5 (1)	4 (1,5)	9 (1,5)
	36–40	1 (0,5)	0 ()	1 (0,5)	2 (0)	0 (0)	2 (0)
	> 40	1 (0,5)	1 (1)	2 (0,5)	0 (0)	3 (1)	3 (0,5)
	**Total**	**103 (49)**	**49 (37)**	**152 (44)**	**195 (45,5)**	**134 (46)**	**329 (46)**
**Clinical**	< 20	0 (0)	0 (0)	0 (0)	0 (0)	0 (0)	0 (0)
	20–25	65 (31)	45 (34)	110 (32)	146 (34)	90 (31)	236 (32,5)
	26–30	25 (12)	28 (21)	53 (15,5)	64 (15,5)	49 (17)	113 (15,5)
	31–35	9 (4)	8 (6)	17 (5)	17 (4)	9 (3)	26 (4)
	36–40	7 (3)	2 (2)	9 (3)	6 (1)	8 (2,5)	14 (2)
	> 40	2 (1)	0 (0)	2 (0,5)	0 (0)	2 (0,5)	2 (0)
	**Total**	**108 (51)**	**83 (63)**	**191 (56)**	**233 (53,5)**	**158 (54)**	**391 (46)**

### Gender discrimination and sexual harassments

**Fig. 1:**
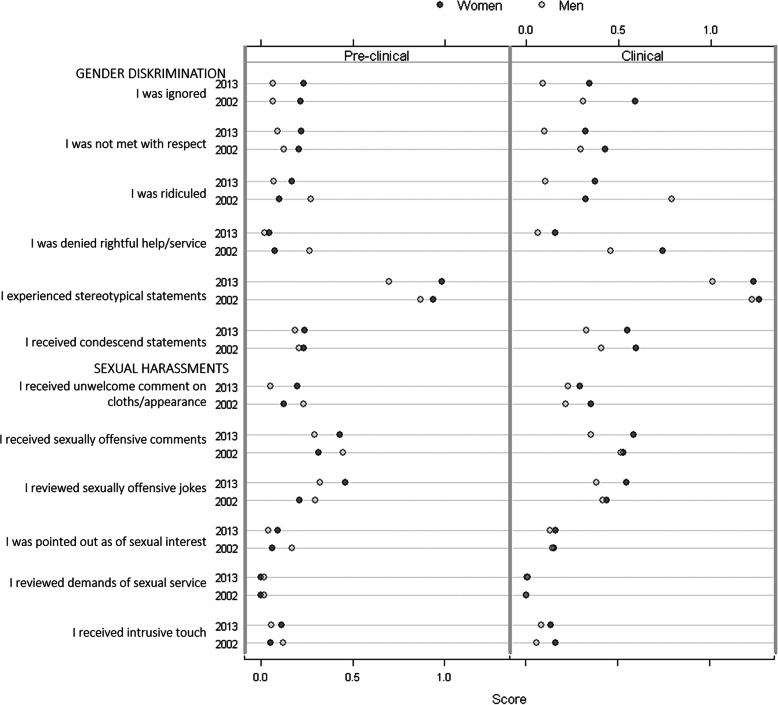
Mean values shows the frequency of exposures to gender discrimination (GD) and sexual harassments (SH) experienced by female and male medical students at pre-clinical and clinical semesters in 2002 and 2013. Scale: Never = 0, Once = 1, More than once = 3

The changes in the prevalence (ratio) of GD and SH in medicals students between 2002 and 2013 are presented in Supplementary Table 2 and summarized in Fig. 1. One student could report one or several types of exposure and indicate its frequency as ‘never,’ ‘once,’ or ‘more than once.’ We also calculated if there was any significant difference between women and men in the ratio of different behaviors.

GD: The prevalence of GD among clinical students decreased between 2002 and 2013. In three types of behaviors such as ‘being ignored,’ ‘being disrespected,’ and ‘being ridiculed,’ the decrease was significantly higher in men compared to women.

In the pre-clinical group, the prevalence of GD increased in females. The prevalence of ‘being disrespected’ more than once doubled in women between 2002 and 2013 (ratio: 2.73 CI: 0.07–114.5). Also, the number of men who experienced this behavior once increased (ratio: 1.67 CI: 0.41–6.73).

SH: Female pre-clinical students reported an increase in the prevalence for five out of six stated behaviors between 2002 and 2013. For three behaviors, the ratio doubled (Supplementary Table 2 and Fig. 1):

- ‘Unwanted comment on my clothes and appearance,’ more than once (ratio: 2.11 CI: 0.35–12.7)
- ‘Sexually offensive comments directed toward me,’ more than once (ratio: 2.17 CI:0.60–7.80)
- ‘Sexually offensive jokes,’ once (ratio 2.00 CI: 0.89–4.51), more than once (ratio: 2.30 CI: 0.60–8.75)
- ‘Pointed out as a sexual object,’ once (ratio: 2.53 CI: 0.41–15.8)

The ratio of several behaviors also increased among the female clinical students. There was a decrease in the ratios of prevalence of SH noted in the male group, in particular among the pre-clinical students. In 2002, there were almost no reports of ‘receiving demands of sexual services.’ These reports, however, increased in numbers in 2013.

### Observing mistreatment of others

The medical students were asked if they had observed other students being mistreated or favored due to gender. The students reported a decrease in observing other students being mistreated and favored (Table 2). However, observations of ‘discrimination’ were reported more often by male pre-clinical students in 2013 than in 2002 (ratio 1.39 CI: 0.57–3.37).

**Table 2:** The medical students in the primary and secondary cohort, divided into pre-clinical and clinical courses as well as gender (female/male), were asked the question: “Did you notice other students being discriminated / favored /receiving unwelcome comments due to her/his gender?” Prevalence (percent) of the medical students who answered ‘yes’ to this question (weighted to represent the total population of medical students during 2002 and 2013, respectively). Ratios presented are the prevalence in 2013 divided by the prevalence in 2002, with corresponding 95% confidence intervals. A ratio > 1 indicates that the condition has an increased prevalence in 2013. The *p*-values result from tests where the null hypotheses are that the ratios are the same between the males and the females. Thus, a *p*-value < 0.5 indicates a statistically significant ratio between the sexes over the time period

Did you notice that other students experienced ......due to her/his gender?	Cohort	Pre-clinical		Clinical	
		Females	Males	Females	Males
(… discrimination …	**2002** (percent)	26.6	10.5	42.6	31.1
	**2013** (percent)	22.4	14.6	41.3	25.0
	Ratio (2013/2002)	0.84 (0.67–1.06)	1.39 (0.57–3.37)	0.97 (0.81–1.17)	0.80 (0.65–1.00)
	*p*-value	0.246		0.244	
… favoritism …	**2002** (percent)	31.6	18.6	66.4	42.8
	**2013** (percent)	22.9	17.8	53.0	33.0
	Ratio (2013/2002)	0.72 (0.61–0.86)	0.95 (0.60–1.52)	0.80 (0.73–0.88)	0.77 (0.65–0.91)
	*p*-value	0.369		0.785	
…. intrusive unwelcome acts … ).	**2002** (percent)	19.7	16.4	27.1	32.6
	**2013** (percent)	20.8	14.7	25.5	20.2
	Ratio (2013/2002)	1.06 (0.75–1.48)	0.89 (0.56–1.44)	0.94 (0.73–1.20)	0.62 (0.52–0.74)
	*p*-value	0.596		0.040	

### The contexts and indicated perpetrators

The students were asked to indicate the attributes of the perpetrator (Table 3). We found that mistreatment of female pre-clinical students increased at all learning contexts between 2002 and 2013; specifically, occurrences in the clinical context were five to seven times as common in 2013. Male pre-clinical students also experienced an increase in mistreatment during clinical training. Clinical students reported a decrease in mistreating behaviors during the theoretical parts of the education.

**Table 3:** The stratification of the individuals reported as perpetrators (other student/supervisor or teacher/medical doctor/ nurse or other medical personnel) of sexual harassments. The medical students from the primary cohort (2002) and the revision cohort (2013) were stratified by pre-clinical and clinical semester and gender (females/males). Prevalence (percent) of the medical students who answered ‘yes’ to this question (weighted to represent the total population of medical students during 2002 and 2013, respectively). Ratios presented are the prevalence in 2013 divided by the prevalence in 2002, with corresponding 95% confidence intervals. A ratio > 1 indicates that the condition has an increased prevalence in 2013. The *p*-values result from tests where the null hypotheses are that the ratios are the same between the females and the males

Semester	Gender	Cohort	Other student who was		Supervisor/teacher who was		Medical doctor who was		Nurse or other medical personnel who was	
			Females	Males	Females	Males	Females	Males	Females	Males
Pre-clinical	**Female**	2002 (percent)	25.2	41.2	22.0	48.2	4.2	8.6	4.2	4.2
		2013 (percent)	28.6	37.9	23.1	29.3	11.4	14.1	7.9	7.5
		Ratio (2013/2002)	1.14 (0.85–1.52)	0.92 (0.77–1.09)	1.05 (0.78–1.41)	0.61 (0.55–0.68)	2.70 (0.48–15.06)	1.65 (0.75–3.60)	1.86 (0.55–6.30)	1.78 (0.55–5.70)
	**Male**	2002 (percent)	36.4	44.0	14.9	19.2	4.7	4.7	5.6	4.2
		2013 (percent)	21.1	21.8	14.4	12.2	6.2	6.8	2.4	1.8
		Ratio (2013/2002)	0.58 (0.49–0.69)	0.50 (0.44–0.56)	0.97 (0.59–1.57)	0.64 (0.48–0.85)	1.33 (0.44–4.04)	1.46 (0.44–4.86)	0.44 (0.29–0.65)	0.44 (0.27–0.70)
		*p*-value	0.001	< 0.001	0.766	0.862	0.191	0.796	0.029	0.037
Clinical	**Female**	2002 (percent)	40.9	54.3	56.3	73.0	51.6	62.3	42.8	38.5
		2013 (percent)	44.0	55.4	39.8	52.1	56.7	74.8	40.1	40.7
		Ratio (2013/2002)	1.08 (0.88–1.32)	1.02 (0.88–1.18)	0.71 (0.64–0.79)	0.71 (0.66–0.77)	1.10 (0.93–1.29)	1.20 (1.04–1.38)	0.94 (0.79–1.11)	1.06 (0.86–1.29)
	**Male**	2002 (percent)	47.5	46.3	42.2	48.2	37.7	42.5	31.5	23.9
		2013 (percent)	40.8	46.9	32.1	34.0	48.3	50.7	35.9	30.4
		Ratio (2013/2002)	0.86 (0.72–1.03)	1.01 (0.82–1.25)	0.76 (0.64–0.91)	0.71 (0.61–0.82)	1.28 (0.95–1.72)	1.19 (0.93–1.54)	1.14 (0.84–1.56)	1.27 (0.85–1.92)
		*p*-value	0.113	0.951	0.615	0.921	0.290	0.955	0.256	0.351

In parallel with those results, there was an increase in medical doctors of both sexes being reported as perpetrators, which was noted for all medical students (Table 3). Pre-clinical female and clinical male students were more often mistreated by nurses or other medical personnel in 2013 than in 2002. The opposite was noted for pre-clinical male students. Reports of other peers as perpetrators were generally consistent but decreased for pre-clinical male students.

## Discussion

In this study, we analyzed the changes in prevalence and the pattern of self-reported experience of gender discrimination (GD), including gender harassment, and sexual harassment (SH) of medical students between 2002 and 2013 at Uppsala University (UU) in Sweden.

In this study we separated SH from other GD as those types of behaviors are the most alarming and must be highlighted [35]. In the presentation of the results, we focus on increased ratios as it is the most important to define what interventions are needed for the future. The main result of our study is that although the prevalence of several examples of GD showed a tendency to decrease between 2002 and 2013, these results are complicated by an increased difference between the sexes, widening the gender gap for prevalence of GD and SH. Alarmingly, the prevalence of SH increased for both women and men, but particularly in pre-clinical settings, where the prevalence of some behaviors doubled. The most severe form of SH, the demand for sexual acts, was uncommon in 2002 but more common in 2013. Based on the fact that SH-experiences increased but not the observations of others being subjected to these behaviors, we can suspect that these interactions occur behind the scenes, making it harder for others to notice.

Our previous studies concluded that the total prevalence of self-reported GD/SH was higher in students during clinical rather than pre-clinical training and affects women more than men [21, 22]. In this study, the graphics in Fig. 1 support these conclusions. It is worrying but not surprising as several other studies and the recent meta-analysis presented similar outcomes [2, 36].

Several studies showed that discriminating behaviors lead to negative consequences for medical students and their professional future [10, 37, 38]. In addition, even observations of other students being mistreated have a negative impact [39]. Therefore, we also analyzed if medical students observed other students being discriminated against, which was common. Pre-clinical male students who between the years became less subjected to GD themselves noticed a higher prevalence of mistreatment of other students, which supports the self-reported increase in experiences among pre-clinical female students.

The reason of increasing reporting of GD/SH in 2013 compared to 2002 is most probably due to raised awareness of those behaviors among students. This can be a result of universities’ attempt to address discriminations, such as curricular changes done at UU in 2006 [18] but also conventional media and culture. Also, social media had increasingly offered information on the topic, even long before #MeToo.

In our opinion rising awareness of gender equality and understanding gender discrimination might contribute to higher response rate in 2013 than in 2002. Also, the distribution during lectures as done in 2013 was probably more effective than using postal service in 2002.

Between 2002 and 2013, the context of mistreatment changed and was less common during the theoretical moments but increased in the clinical context, most noted in the pre-clinical group and especially in the female pre-clinical group. In this group, the occurrence of mistreatment during clinical training increased five times by female supervisors and seven times by male supervisors between 2002 and 2013. This is probably partly explained by changes in the educational program at UU, where pre-clinical students in the 2013 cohort have had regular clinical rotations in primary care since the first semester. Out of all the clinical supervisors, medical doctors were most often identified as the perpetrator in 2013.

Although male students’ experiences of GD in general changed for the better, reports on medical doctors as perpetrators increased also in this group. Evidently, these curricular interventions fail to reach the clinical environment. When mistreatment is not addressed properly, studies suggest that students may incorporate the bad norms and attitudes of the teaching physicians as part of the hidden curriculum [40, 41]. When the teaching physician is being discriminatory, it may cause a negative spiral of these behaviors. The acceptance of mistreatment may increase and even worse, people who suffered from mistreatment often become perpetrators themselves [42].

### Strengths and limitations

Our research benefited from two independent cohorts of medical students. Because of the large number of participants and high response rate, our results may be generalizable to other medical schools with similar educational structure. Another advantage is the use of previously validated behavioristic questions, which helped to decrease the risk of bias.

There are however limitations to this study. It is cross-sectional with no control group. The questionnaires use closed-ended questions, where respondents are not able to clarify their choice or verify if the interpretation was the one intended. With self-reports, there is always a perception bias and participants offer their subjective experiences. The examples of GD/SH may be differently interpreted between individuals and groups, and there may be a risk for result bias. The study evaluated only the experience of medical students (victim’s perspective). Some aspects that may impact the answers were not considered in the study such as other minority identities reflecting other grounds of discrimination and social status of the participant. Another limitation may be that the authors analyzed data from their own institution.

### Study implications

With this study, we want to show that medical students experience GD/SH during their medical education. Moreover, the context of those experiences is often the clinical environment, which can be hard for universities to monitor and change. Efforts by universities to detect gender discrimination with the currently used instruments such as course evaluations might not be adequate as underreporting is a known problem [43–45], and response rates are usually low. Recurrent mistreatment of medical students by faculty staff was showed to be of the driving forces of burnout, and psychiatric disorders [46, 47]. Evidence-based strategies to decrease GD/SH involved education of staff and students including knowledge of student’s rights, proactive encouragement of reporting, and regular monitoring of discriminatory behaviors [2, 7, 8, 39]. Also, teaching on gender equality may play a pivotal role in bridging the gap from gender bias to gender awareness [48]. Change might depend on educating and promoting reflexive abilities and well-being among clinical supervisors [49]. All this to align the formal curriculum including confident engaging in learning activates supported by teaching stuff with the informal or hidden one, thus creating a good learning environment for students.

## Conclusions

Our study may be the first study documenting the prevalence and contextual changes of GD/SH among medical students over such a long time as 11 years. The study shows that the prevalence of GD/SH in many cases decreased in male students and increased, particularly among female pre-clinical students, widening the gender gap for prevalence of GD/SH. Alarmingly, the prevalence of SH increased, especially among female students. The exposure to GD/SH increased mainly in the clinical context; nonetheless, this should not be tolerated by universities and hospitals. Medical doctors are often indicated as perpetrators, and curricular interventions to decrease GH/SH seldom reach this part of the education. In order to improve awareness of gender equality in health care workers, the interventions countering GD/SH should be considered an essential investment improving health care quality [50]. For example, a mandatory course on gender equality could be a part of medical doctors’ professional education. Further, we will repeat this investigation with medical students at Uppsala University in 2020. We hope for a “#MeToo effect” that improves awareness of GD/SH and put end to the existing mistreatments.

## Supplementary Information

**Additional file 1: Table S1**. The questions used in the study and selected from the questionnaire. **Table S2**. The frequency, defined as once or at least twice of different examples of self-reported gender discrimination and sexual harassments, was calculated. The 2002 and 2013 cohorts were stratified into pre-clinical and clinical students and gender in cohort 2002 and 2013. Prevalence (percent) of the medical students who answered ‘yes’ to this question (weighted to represent the total population of medical students during 2002 and 2013, respectively). Ratios presented are the prevalence in 2013 divided by the prevalence in 2002, with corresponding 95% confidence intervals. A ratio > 1 indicates that the condition has an increased prevalence in 2013.

## Data Availability

The data is available in corresponding author data base and is available for all coauthors. It can be shown if requested.

## Abbreviations

GD: Gender discrimination
SH: Sexual harassment
UU: Uppsala University
CI: Confidence interval
#MeToo: Or the MeToo international movement against sexual harassment and sexual abuse on social media
