# Supplementary material for: Medical students’ self-reported gender discrimination and sexual harassment over time
Source: BMC Med Educ. 2020 Dec 10;20:503. doi: 10.1186/s12909-020-02422-9 (PMC7731624; doi:10.1186/s12909-020-02422-9)
Supplement: Supplementary file 1 — Additional file 1: Table S1. The questions used in the study and selected from the questionnaire. Table S2. The frequency, defined as once or at least twice of different examples of self-reported gender discrimination and sexual harassments, was calculated. The 2002 and 2013 cohorts were stratified into pre-clinical and clinical students and gender in cohort 2002 and 2013. Prevalence (percent) of the medical students who answered ‘yes’ to this question (weighted to represent the total population of medical students during 2002 and 2013, respectively). Ratios presented are the prevalence in 2013 divided by the prevalence in 2002, with corresponding 95% confidence intervals. A ratio > 1 indicates that the condition has an increased prevalence in 2013. [file 12909_2020_2422_MOESM1_ESM.docx]

**Supplementary Table 1:** The questions used in the study and selected from the questionnaire.

| Questions / answers |
| --- |
| Please select the correct answers?  Semester: 1 2 3 4 5 6 7 8 9 10 11  Sex: female male  Age: < 20 20-25 26-30 31-35 36-40 > 40 years old |
| Please indicate if you have been exposed to any of the following forms of mistreatment and please indicate the frequency as never, once or at least twice? Please note that it must have happened during your medical studies!  I have experienced………due to my gender.   - I was ignored - I was not met with respect - I was ridiculed - I was denied rightful help/services - I experienced stereotypical statements - I received condescended comments - I received unwelcome comments on clothes/appearance - I received sexually offensive comments - I was pointed out as of sexual interest - I experienced intrusive touch - I received sexually offensive jokes - I received demands of sexual services   In what contexts did this happen?   - Lectures - Practical moments - Clinical practice   Who mistreated you? Indicate if the person was a female or male?   - Other student - Lecturer/teacher - Medical doctor - Nurse or other medical personnel |

**Supplementary Table 2:** The frequency, defined as once or at least twice of different examples of self-reported gender discrimination and sexual harassments, was calculated. The 2002 and 2013 cohorts were stratified into pre-clinical and clinical students and gender in cohort 2002 and 2013. Prevalence (percent) of the medical students who answered ‘yes’ to this question (weighted to represent the total population of medical students during 2002 and 2013, respectively). Ratios presented are the prevalence in 2013 divided by the prevalence in 2002, with corresponding 95% confidence intervals. A ratio > 1 indicates that the condition has an increased prevalence in 2013.

| **Types of behaviour** | **Cohort** | **Pre-clinical** | | | | **Clinical** | | | |
| --- | --- | --- | --- | --- | --- | --- | --- | --- | --- |
|  |  | **Frequency** | | | | **Frequency** | | | |
|  |  | **Once** | | **At least twice** | | **Once** | | **At least twice** | |
|  |  | Females | Males | Females | Males | Females | Males | Females | Males |
| **GENDER DISCRIMINATION** | | | | | | | | | |
| **Being ignored** | 2002 (percent) | 15.8 | 6.7 | 3.0 | 0.0 | 45.7 | 21.5 | 6.6 | 4.7 |
|  | 2013 (percent) | 15.7 | 6.7 | 3.9 | 0.0 | 32.4 | 8.7 | 1.0 | 0.0 |
|  | Ratio (2013/2002) | 0.99 (0.68–1.46) **↓** | 1.00 (0.44–2.29) | 1.30 (0.42–4.03) **↑** | - | 0.71 (0.62–0.81) **↓** | 0.41 (0.35–0.48) **↓** | 0.15 (0.13–0.16) **↓** | -**↓** |
|  | p-value | 0.979 | | - | | 0.004* | | - | |
| **Being disrespected** | 2002 (percent) | 18.4 | 5.5 | 1.3 | 3.4 | 30.3 | 24.0 | 6.1 | 2.6 |
|  | 2013 (percent) | 15.2 | 9.2 | 3.4 | 0.0 | 28.6 | 9.8 | 1.8 | 0.0 |
|  | Ratio (2013/2002) | 0.83 (0.63–1.09) **↓** | 1.67 (0.41–6.73) **↑** | 2.73 (0.07–114.5) **↑** | -**↓** | 0.94 (0.75–1.19) **↓** | 0.41 (0.35–0.48) **↓** | 0.29 (0.24–0.35) **↓** | -**↓** |
|  | p-value | 0.004* | | - | | 0.004* | | - | |
| **Being ridiculed** | 2002 (percent) | 10.7 | 4.0 | 5.5 | 0.0 | 39.5 | 25.6 | 15.3 | 3.6 |
|  | 2013 (percent) | 9.3 | 3.8 | 1.9 | 1.6 | 18.6 | 7.4 | 2.2 | 0.0 |
|  | Ratio (2013/2002) | 0.87 (0.58–1.29) **↓** | 0.95 (0.35–2.59) **↓** | 0.34 (0.26–0.44) **↓** | -**↓** | 0.47 (0.42–0.52) **↓** | 0.29 (0.26–0.33) **↓** | 0.14 (0.13–0.15) **↓** | -**↓** |
|  | p-value | 0.248 | | - | | <0.001* | | - | |
| **Denied rightful help/services** | 2002 (percent) | 0.0 | 0.0 | 3.9 | 13.4 | 33.5 | 24.6 | 20.3 | 10.5 |
|  | 2013 (percent) | 3.2 | 2.4 | 0.8 | 0.0 | 11.6 | 4.5 | 2.1 | 0.9 |
|  | Ratio (2013/2002) | -**↑** | -**↑** | 0.21 (0.15–0.30) **↓** | -**↓** | 0.35 (0.28–0.42) **↓** | 0.18 (0.16–0.21) **↓** | 0.11 (0.10–0.12) **↓** | 0.09 (0.08–0.10) **↓** |
|  | p-value | - | | - | | 0.186 | | 0.810 | |
| **Experiencing stereotypical statements** | 2002 (percent) | 44.9 | 40.7 | 24.3 | 22.8 | 41.1 | 42.8 | 42.6 | 39.7 |
|  | 2013 (percent) | 40.4 | 40.1 | 28.7 | 14.6 | 44.1 | 39.8 | 39.6 | 30.4 |
|  | Ratio (2013/2002) | 0.90 (0.77–1.06) **↓** | 0.99 (0.76–1.28) **↓** | 1.18 (0.87–1.61) **↑** | 0.64 (0.48–0.84) **↓** | 1.07 (0.88–1.32) **↑** | 0.93 (0.76–1.13) **↓** | 0.93 (0.79–1.10) **↓** | 0.77 (0.64–0.92) **↓** |
|  | p-value | 0.592 | | 0.010* | | 0.314 | | 0.197 | |
| **Receiving condescended comments** | 2002 (percent) | 13.9 | 11.2 | 4.8 | 4.8 | 34.5 | 29.7 | 12.4 | 5.3 |
|  | 2013 (percent) | 12.2 | 13.0 | 5.8 | 2.8 | 35.4 | 24.1 | 9.6 | 4.1 |
|  | Ratio (2013/2002) | 0.87 (0.62–1.22) **↓** | 1.16 (0.55–2.48) **↑** | 1.21 (0.54–2.74) **↑** | 0.58 (0.31–1.11) **↓** | 1.02 (0.83–1.28) **↑** | 0.81 (0.64–1.03) **↓** | 0.78 (0.57–1.07) **↓** | 0.76 (0.44–1.33) **↓** |
|  | p-value | 0.492 | | 0.231 | | 0.195 | | 0.969 | |
| **SEXUAL HARASSMENT** | | | | | | | | | |
| **Receiving unwelcome comments on clothes/appearance** | 2002 (percent) | 8.0 | 19.2 | 2.3 | 2.0 | 27.1 | 12.3 | 3.9 | 4.4 |
|  | 2013 (percent) | 10.4 | 3.7 | 4.8 | 0.9 | 20.1 | 14.7 | 4.5 | 3.9 |
|  | Ratio (2013/2002) | 1.30 (0.65–2.61) **↑** | 0.19 (0.17–0.22) **↓** | 2.11 (0.35–12.7) **↑** | 0.43 (0.21–0.87) **↓** | 0.74 (0.61–0.90) **↓** | 1.19 (0.70–2.04) **↑** | 1.15 (0.50–2.63) **↑** | 0.89 (0.40–1.96) **↓** |
|  | p-value | 0.002* | | 0.087 | | 0.121 | | 0.660 | |
| **Receiving sexually offensive comments** | 2002 (percent) | 22.0 | 28.4 | 4.7 | 8.0 | 31.4 | 33.9 | 10.5 | 8.7 |
|  | 2013 (percent) | 22.5 | 22.6 | 10.2 | 3.4 | 37.7 | 26.9 | 10.2 | 3.9 |
|  | Ratio (2013/2002) | 1.02 (0.76–1.38) **↑** | 0.80 (0.60–1.05) **↓** | 2.17 (0.60–7.80) **↑** | 0.42 (0.29–0.62) **↓** | 1.20 (0.92–1.58) **↑** | 0.79 (0.64–0.99) **↓** | 0.98 (0.65–1.49) **↓** | 0.46 (0.34–0.61) **↓** |
|  | p-value | 0.279 | | 0.010* | | 0.022 | | 0.045* | |
| **Receiving sexually offensive jokes** | 2002 (percent) | 10.3 | 20.1 | 5.5 | 4.9 | 27.7 | 28.8 | 7.8 | 6.3 |
|  | 2013 (percent) | 20.6 | 23.6 | 12.6 | 4.3 | 33.0 | 31.5 | 10.7 | 3.3 |
|  | Ratio (2013/2002) | 2.00 (0.89–4.51) **↑** | 1.18 (0.69–2.00) **↑** | 2.30 (0.60–8.75) **↑** | 0.87 (0.34–2.25) **↓** | 1.19 (0.88–1.61) **↑** | 1.09 (0.79–1.51) **↑** | 1.37 (0.70–2.69) **↑** | 0.52 (0.35–0.75) **↓** |
|  | p-value | 0.095 | | 0.089 | | 0.664 | | 0.029* | |
| **Being pointed out as of sexual interest** | 2002 (percent) | 3.0 | 9.7 | 1.5 | 3.5 | 7.8 | 10.5 | 3.6 | 1.7 |
|  | 2013 (percent) | 7.6 | 3.9 | 0.8 | 0.0 | 10.3 | 8.6 | 2.8 | 2.0 |
|  | Ratio (2013/2002) | 2.53 (0.41–15.8) **↑** | 0.40 (0.29–0.55) **↓** | 0.50 (0.26–0.98) **↓** | -**↓** | 1.31 (0.68–2.51) **↑** | 0.82 (0.53–1.26) **↓** | 0.78 (0.43–1.43) **↓** | 1.16 (0.21–6.48) **↑** |
|  | p-value | 0.025* | | - | | 0.220 | | 0.681 | |
| **Receiving demands of sexual services** | 2002 (percent) | 0.0 | 0.2 | 0.0 | 0.0 | 0.0 | 0.0 | 0.0 | 0.0 |
|  | 2013 (percent) | 0.0 | 0.0 | 0.0 | 1.0 | 0.4 | 0.9 | 0.0 | 0.0 |
|  | Ratio (2013/2002) | - | - **↓** | - | -**↑** | -**↑** | -**↑** | - | - |
|  | p-value | - | | - | | - | | - | |
| **Experiencing intrusive touch** | 2002 (percent) | 5.2 | 6.4 | 0.0 | 2.9 | 12.5 | 5.6 | 1.5 | 0.0 |
|  | 2013 (percent) | 9.8 | 3.7 | 0.8 | 1.0 | 10.2 | 8.1 | 1.4 | 0.0 |
|  | Ratio (2013/2002) | 1.87 (0.61–5.72) **↑** | 0.58 (0.37–0.91) **↓** | -**↑** | 0.35 (0.22–0.57) **↓** | 0.82 (0.59–1.14) **↓** | 1.44 (0.52–4.03) **↑** | 0.91 (0.29–2.88) **↓** | - |
|  | p-value | 0.035 * | | - | | 0.256 | | - | |
